# Transcriptomic evidence of CD8⁺ T-cell exhaustion-like phenotype and mitochondrial impairment underlying immune dysregulation in feline chronic gingivostomatitis

**DOI:** 10.1038/s41598-026-50658-0

**Published:** 2026-05-06

**Authors:** Maria Soltero-Rivera, Patrawin Wanakumjorn, Yihong Chen, Samantha Barnum, Luis Diego Castillo Charpentier, Boaz Arzi, Natalia Vapniarsky Arzi, Amir Kol

**Affiliations:** 1 Department of Surgical and Radiological Sciences, University of California, Davis, USA; 2 Department of Pathology, Microbiology & Immunology, University of California, Davis, USA; 3 Department of Medicine and Epidemiology, University of California, Davis, USA

**Keywords:** Feline, Oral medicine, Stomatitis, Transcriptomics, CD8 + lymphocytes, Mesenchymal stromal cells, Exhaustion, Diseases, Immunology

## Abstract

Feline chronic gingivostomatitis (FCGS) is a debilitating oral disease characterized by immune dysregulation and chronic inflammation. We hypothesized that CD8 + T cells from FCGS cats exhibit exhaustion features with suppressed mitochondrial pathways, and that mesenchymal stromal cell (MSC) therapy post-extractions might restore these programs. RNA sequencing was performed on peripheral CD8 + T cells from cats with active FCGS before (disease group, D) and after (treated group, M) clinical remission following full-mouth extractions and MSC therapy, with specific-pathogen-free cats as controls (control group, C). CD8 + T cells from active disease displayed terminal effector differentiation and exhaustion-like signatures, including upregulation of cytotoxic markers (GZMB, GZMK, GZMA), differentiation markers (KLRG1, IL18R1/IL18RAP), and exhaustion-associated genes (EOMES, CD244, TNFSF10, CCR5, PRDM1, RGS16). Gene set enrichment analysis confirmed exhaustion-like CD8 + T-cell phenotype enrichment in active disease, which resolved after treatment. Pathway analysis revealed marked downregulation of mitochondrial respiratory chain components, ATP synthesis, and protein import pathways in active FCGS, with partial post-treatment resolution. Immunofluorescence of draining lymph nodes showed significantly increased CTLA-4 + CD3+ T cells in both FCGS groups versus controls, suggesting persistent immunoregulatory signaling despite clinical improvement. These findings identify overlapping T-cell exhaustion and mitochondrial dysfunction-associated transcriptomic signatures in FCGS, supporting therapeutic strategies targeting immune-metabolic pathways.

## Introduction

The fate of CD8⁺ T cells plays a central role in shaping immune responses to chronic viral infections, cancers, and persistent inflammatory conditions^1–3^. In these contexts, sustained antigen stimulation often leads to T cell exhaustion, a dysfunctional state marked by reduced effector function, increased inhibitory receptor expression, altered epigenetic architecture, and loss of long-term memory potential^4–9^. While considerable progress has been made in characterizing the transcriptional programs underlying exhaustion, mitochondrial dysfunction has recently emerged as a central mechanistic driver of this state^6,10–16^. Exhausted CD8⁺ T cells exhibit impaired oxidative phosphorylation, reduced mitochondrial mass and membrane potential, increased reactive oxygen species, and a collapse of metabolic flexibility, features increasingly viewed as both a consequence and a cause of dysfunctional immune responses^14^.

Mitochondria serve as essential regulators of immune cell fate, integrating environmental signals and energetic demands to control proliferation, persistence, and cytokine production^17^. In CD8⁺ T cells, functional mitochondria support the transition from effector to memory states, sustain ATP generation under metabolic stress, and regulate redox signaling necessary for survival and function^18^. Conversely, mitochondrial impairment has been shown to skew cells toward terminal exhaustion, with studies in murine models of chronic viral infection and cancer demonstrating that restoring mitochondrial function can reinvigorate T cell responses and improve immunotherapy efficacy^14,15,18–21^. Despite these advances, most of our understanding derives from engineered or artificial model systems. There remains a critical need for spontaneous, immunocompetent models that recapitulate the complexity of chronic mucosal inflammation and immune dysfunction in natural disease.

Feline chronic gingivostomatitis (FCGS) represents a uniquely valuable model in this context. FCGS is a painful, progressive inflammatory disease of the feline oral mucosa associated with chronic antigenic stimulation, T cell–rich infiltrates, and poor response to conventional therapies^22^. CD8⁺ T cells are a dominant population in affected tissues, and prior studies suggest they may contribute to both tissue damage and immune dysregulation^23,24^. Intriguingly, we and others have shown that mesenchymal stromal cell (MSC) therapy, delivered systemically, results in significant clinical improvement in FCGS, including sustainable resolution of oral lesions and systemic immune rebalancing^25–29^. In our previous work using a related feline model of chronic coronavirus infection (feline infectious peritonitis, FIP), we demonstrated that MSC treatment not only modulated inflammation but also rescued key pathways associated with mitochondrial bioenergetics and metabolic homeostasis^30^. These findings suggest that MSCs may exert therapeutic effects in part through restoration of mitochondrial function in immune effector cells, including exhausted CD8⁺ T cells.

Despite these promising observations, the potentially exhausted state and mitochondrial function of CD8⁺ T cells in FCGS have never been systematically defined, nor has the mechanism by which MSC therapy might influence T cell mitochondrial metabolism in this setting been elucidated. Given the importance of mitochondrial health in shaping immune fate, this represents a critical gap in our understanding, both for veterinary medicine and for the development of comparative models of chronic immune dysfunction. We hypothesized that CD8 + T cells from cats with FCGS exhibit features of exhaustion accompanied by suppression of mitochondrial bioenergetic pathways, and that MSC treatment might partially restore these transcriptional programs.

To test this, we performed RNA sequencing of sorted CD8⁺ T cells from cats with active FCGS, healthy controls, and cats with inactive FCGS that were treated with MSC and were in a state of long-term remission at the time of sample collection. We further performed confocal immunofluorescence microscopy studies on mandibular lymph node and oral mucosal tissues from these 3 cat populations.

## Results

### Chronic viral infections are common in cats with FCGS

Building on previous research, the viral infection status including feline calicivirus (FCV), feline leukemia virus (FeLV), and feline immunodeficiency virus (FIV) was determined for cats with FCGS when samples obtained prior to treatment were available^31–33^. Among the cats diagnosed with FCGS and tested for viral infections, 71% (12 out of 17) tested positive for FCV. One tested positive FIV (6.3%, 1 out of 16) and no patients tested positive for FeLV. In total, 13 of 17 cats were affected by at least one viral infection.

### Sequencing

**Fig. 1:**
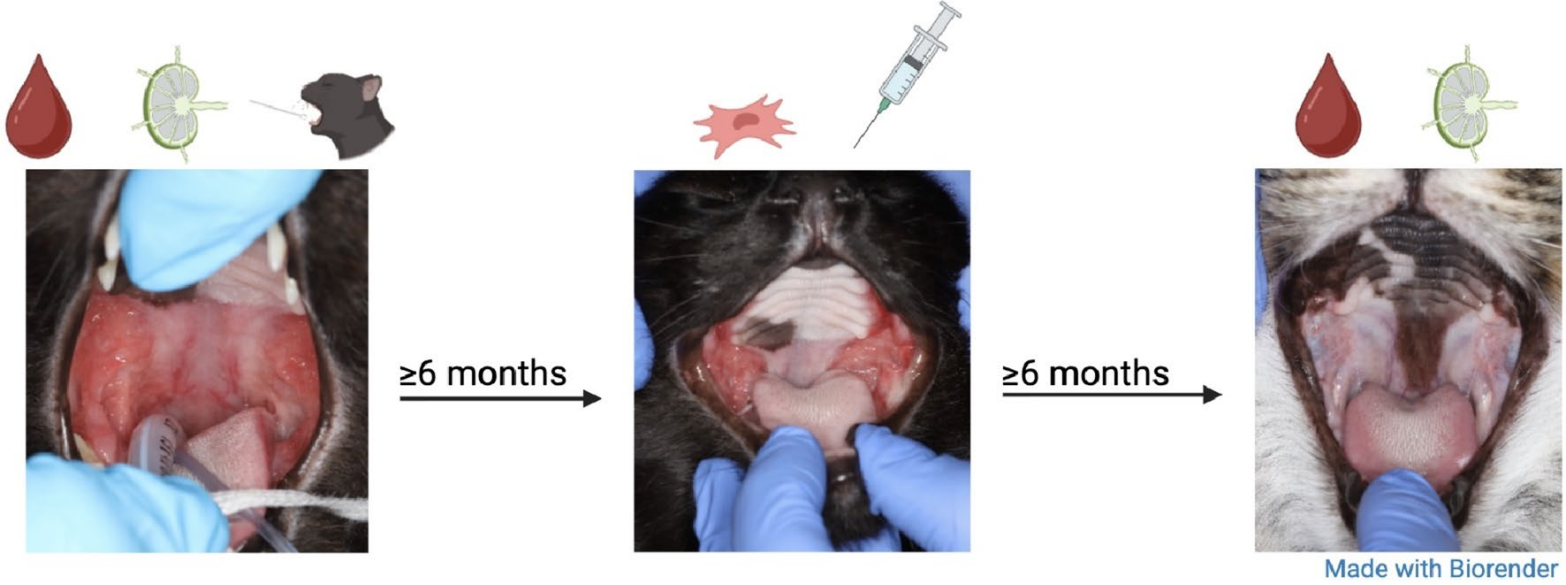
Experimental timeline. Clinical timeline of cats with FCGS undergoing full mouth extractions and mesenchymal stromal cell therapy (MSC). Baseline oral and systemic samples were collected, followed by administration of allogeneic, adipose derived MSCs. Oral examinations were repeated at ≥ 6-month intervals to assess mucosal healing and monitor immunologic and clinical response.

We collected blood from cats with active FCGS before treatment (D; *n* = 8) and cats with inactive FCGS after treatment (M; *n* = 4) which included full mouth extractions (FME) and MSC treatment. We also collected blood from 10 healthy control cats with no evidence of FCGS. CD8 + T cells were enriched via magnetic labeling and bulk RNAseq was used to characterize the transcriptomic landscape of these cells. The average (± SD) number of raw reads per sample before quality control was 7.9 ± 3.0 million. After adapter trimming with Cutadapt and alignment to the feline reference genome (*F.catus_Fca126_mat1.0 genome GenBank assembly GCA_018350175.1*) using STAR, reads were deduplicated using UMI-tools. Post-filtering, the final dataset contained an average of 2.0 ± 0.4 million reads per sample, corresponding to 25.3 ± 5.1% of the original input. The samples showed consistent quality with mean Phred scores > 40 across all base positions. In total, 150 million reads were sequenced across all 19 samples, with an average of 48.6 ± 14.0% successfully assigned to genes using featureCounts. Following filterByExpr criteria, 11,410 expressed genes were retained for downstream differential expression analysis (Fig. 1).

### Transcriptomic signatures of CD8⁺ T-cell exhaustion-like, transcriptional remodeling, and mitochondrial impairment in FCGS

**Fig. 2:**
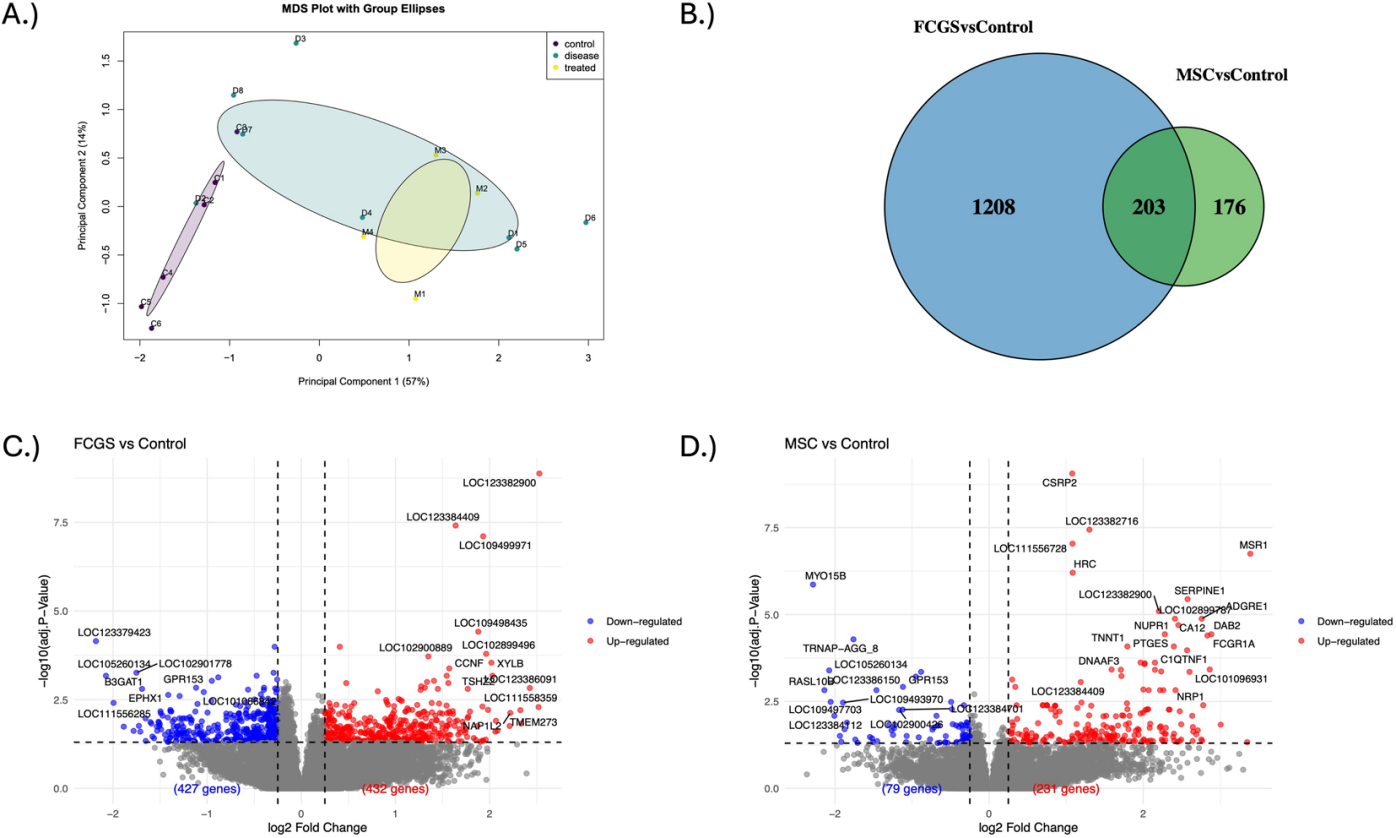
Transcriptomic and pathway-level characterization of CD8⁺ T cells from healthy controls (**C**), untreated FCGS cats (**D**), and MSC-responsive cats (M). (**A**) Multidimensional scaling (MDS) with 95% confidence ellipses demonstrates clear separation of control and FCGS groups, with treated cats occupying an intermediate transcriptional space. (**B**) Venn diagram showing differentially expressed genes (DEGs) unique to D vs. C and M vs. C, as well as DEGs shared across both comparisons, includes all genes that were statistically significant (FDR-adjusted *P* < 0.05) in each contrast. (**C**–**D**) Volcano plots highlighting significantly up- and down-regulated genes for D vs. C and M vs. C with significance defined by both FDR < 0.05 and |log2FC| > 0.25, illustrating transcriptional activation, immune dysregulation, and treatment-associated shifts. Notes: Genes with small but statistically significant changes appear in the Venn diagram but are not shown in the volcano plots, resulting in fewer genes displayed in the volcano panels. Genes shown as overlapping in the Venn diagram represent the same genes across contrasts, but their fold-change values are calculated separately for each comparison and may differ in both direction and magnitude.

A multidimensional scaling (MDS) plot demonstrated a clear and distinct clustering pattern of the healthy controls (C) vs. individuals that were affected by FCGS (D and M). Nevertheless, there were no clear clustering patterns separating the active disease (D) and remission (M) groups. (Fig. 2A). Out of the 11,410 genes aligned with the F.catus_Fca126_mat1.0 genome, 1,208 genes were statistically significantly differentially regulated (adj. P-value < 0.05) in the FCGS group (D) compared to controls (C). The post-treatment group (M) showed 176 significant genes compared to the control (C) (Fig. 2B).

FCGS CD8⁺ T cells exhibited robust transcriptional dysregulation, with the greatest induction observed for LOC123382900, LOC123384409, LOC109499971, LOC109498435, LOC102908946, CCNF, XYLB, and NAP1L3, while LOC123379423, LOC105260134/LOC102901778, B3GAT1, GPR153, EPHX1, and LOC111556225 represented the most significantly suppressed genes compared with healthy controls (Fig. 2C). Up-regulated CCNF, XYLB, and NAP1L3 collectively suggest enhanced cell-cycle regulation, metabolic reprogramming, and chromatin remodeling, molecular features consistent with chronic activation and transcriptional stress in FCGS CD8⁺ T cells. Down-regulated B3GAT1, GPR153, and EPHX1 indicate impaired leukocyte trafficking, disrupted modulatory signaling, and reduced oxidative stress-detoxifying capacity, collectively consistent with a dysfunctional and metabolically strained phenotype in FCGS CD8⁺ T cells.

CD8⁺ T cells from treated cats displayed a distinct transcriptional profile, with the strongest induction observed for MSR1, CSRP2, LOC123382716, LOC111556728, PTGES, C1QTNF1, SERPINE1, and NUPR1, while MYO15B, TRNAP-AGG_8, LOC105260134, GPR153, LOC109493970, LOC123386150, LOC109497703 and LOC123384012 were among the most significantly down-regulated genes compared with healthy controls (Fig. 2D). Compared with healthy controls, treated cats display moderate transcriptional activation marked by up-regulation of genes involved in inflammatory modulation (PTGES, SERPINE1), tissue remodeling (MSR1), metabolic–immune crosstalk (C1QTNF1), and cellular stress adaptation (NUPR1, HRC). In contrast, down-regulated genes include components of translational machinery and signaling regulators (MYO15B, GPR153), indicating decreased cytoskeletal activation and lower signaling burden. Collectively, these changes reflect a shift from the cytotoxic/exhaustion-like signature seen in active FCGS toward a remodeled, less inflammatory, and potentially resolving immune state following therapy.

### Exhaustion-associated transcriptional reprogramming and naïve-to-effector shifts in CD8⁺ T cells from FCGS cats

**Fig. 3:**
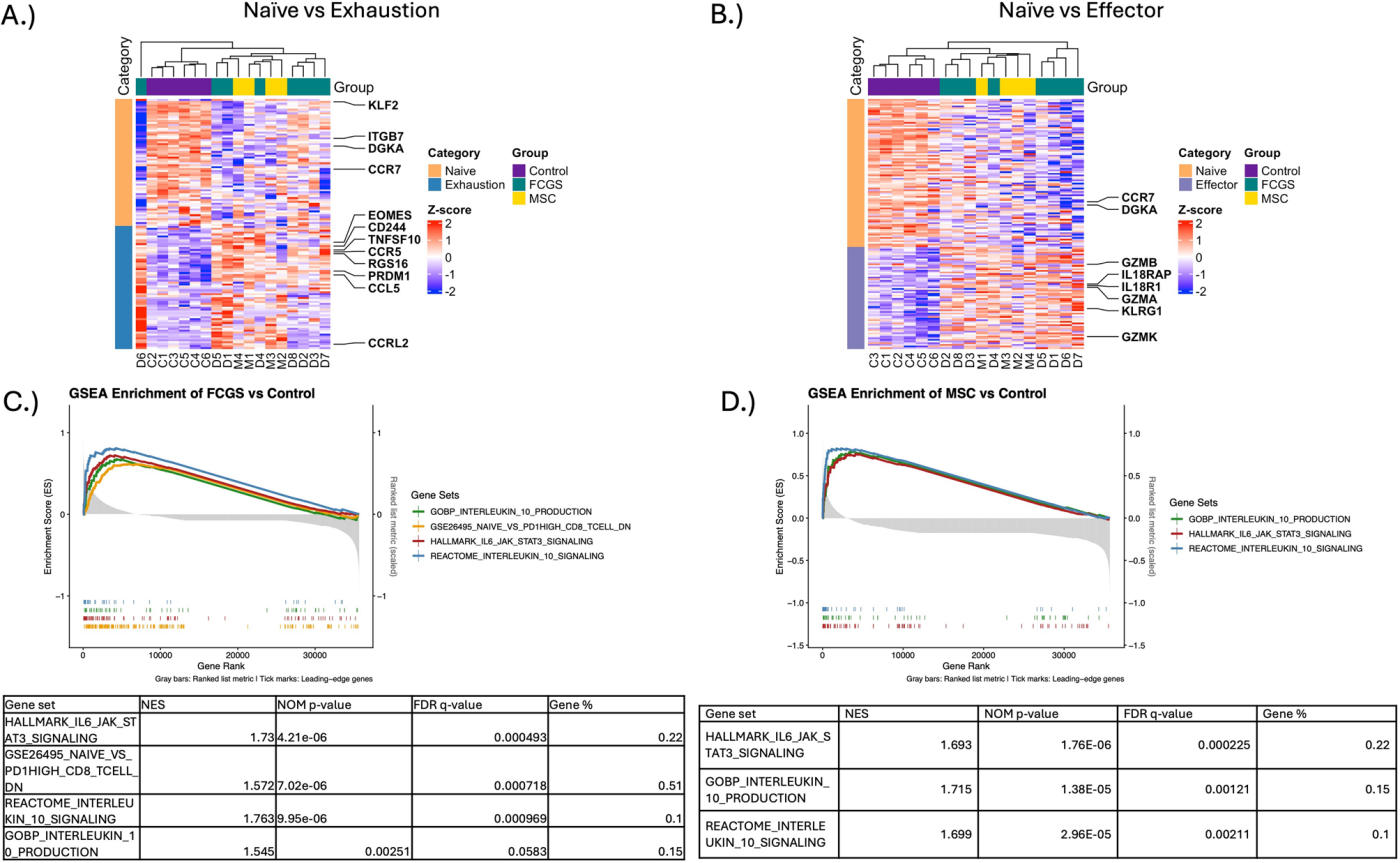
Comparative Profiling of Naïve, Effector, and Exhausted CD8⁺ T-Cell Programs in FCGS and MSC-Responsive Cats. CD8⁺ T-cell state characterization in FCGS and treatment-responsive cats. (**A**–**B**) Heatmaps comparing naïve versus exhaustion and naïve versus effector signatures show that FCGS CD8⁺ T cells shift away from a naïve phenotype and toward exhaustion-like and effector-like transcriptional programs, with treated cats exhibiting an intermediate pattern. Key genes distinguishing these states (e.g., KLF2, CCR7, EOMES, TNFSF10, GZMB, KLRG1) are highlighted. (**C**–**D**) Gene set enrichment analysis (GSEA) for FCGS vs. control and MSC vs. control identifies consistent activation of IL-6/JAK/STAT3 signaling, IL-10 signaling, and PD-1^high CD8⁺ T-cell exhaustion pathways. (**E**–**F**) Statistical summaries of enriched gene sets include normalized enrichment scores (NES), nominal p values, FDR q values, and percentages of contributing genes, confirming robust enrichment of immune-regulatory and cytokine signaling pathways.

Since most of the cats with FCGS had evidence of chronic viral infection (76%), we evaluated the CD8 + T cell transcriptome for exhaustion related signatures. The transcriptomes of CD8 + T enriched cells in FCGS cases were compared before and after treatment as well as with healthy controls. In the Naïve versus Exhaustion comparison (Fig. 3A), the control group showed upregulation of genes expressed by naïve cells such as KLF2, CCR7, ITGB7 and DGKA whereas cats with FCGS (both D and M groups) showed mostly downregulation of these genes. On the other hand, the two FCGS groups showed upregulation of exhaustion related genes including EOMES, CCRL2, TNFSF10, CD244, CCR5, RGS16, PRDM1 and CCL5 while the control group was characterized by downregulation of these. Among the genes upregulated in CD8 + T cells from FCGS cats, EOMES and RGS16 are intrinsic drivers of exhaustion^34,35^ and PRDM1 (Blimp-1) promotes terminal differentiation^36^. Interestingly, DGKA which inhibits the DAG/ERK pathway, contributing to exhaustion was upregulated in control patients^37^.

Gene set enrichment analysis revealed strong activation of exhaustion-associated cytokine pathways in CD8⁺ T cells from cats with FCGS (Fig. 3C). In the FCGS (D) vs. control comparison, the IL-6/JAK/STAT3 signaling hallmark gene set showed the highest, though modest, enrichment (NES = 1.734, NOM *p* = 2.1 × 10⁻⁶, FDR q = 4.9 × 10⁻⁴), followed by the PD-1^high CD8⁺ T-cell exhaustion signature (NES = 1.572, NOM *p* = 7.02 × 10⁻⁶, FDR q = 7.18 × 10⁻⁴). Additional enrichment of IL-10 signaling (NES = 1.545, NOM *p* = 0.00251, FDR q = 0.0583) and interleukin-10–related pathways further supported a shift toward an exhaustion-like transcriptional program in FCGS CD8⁺ T cells.

In the Naïve vs. Effector comparison (Fig. 3B), the naive genes are predominantly highly expressed in the healthy controls. They are generally lowly expressed in the FCGS before (D) and after (M) treatment. The opposite effect is seen in the effector gene cluster. Genes such as GZMA, GZMB and GZMK as well as IL18R, IL18RAP and KLRG1 were upregulated in the FCGS groups and downregulated in the control group. A heatmap highlights that T cells in FCGS (D) show a significant shift away from a naïve phenotype towards an effector phenotype. The presence of granzymes suggests that these cells have differentiated into an effector-like phenotype^38^. IL18R, IL18RAP (Interleukin-18 Receptor alpha and accessory protein) form the receptor for IL-18, a pro-inflammatory cytokine that promotes Th1 responses and enhances the cytotoxicity of cytotoxic T and NK cells. This indicates that T cells in FCGS are exposed to and highly responsive to pro-inflammatory signals, potentially trapping them in a cycle of chronic activation that ultimately drives exhaustion^39^. KLRG1 (Killer Cell Lectin Like Receptor G1) is an inhibitory receptor expressed on highly differentiated, senescent, or terminally exhausted T cells. This marker is often co-expresses with inhibitory receptors (like PD-1) on terminally exhausted T cells^40^. The high expression of KLRG1 strongly suggests that the CD8 + T cells in FCGS are not merely active but are in a state of terminal differentiation and likely exhaustion-like/senescence.

Following therapy, these immune-regulatory pathways remained significantly enriched but with reduced magnitude (Fig. 3D). In the treatment vs. control comparison, IL-6/JAK/STAT3 signaling continued to display modest enrichment (NES = 1.693, NOM *p* = 1.76 × 10⁻⁶, FDR q = 2.25 × 10⁻⁴), along with IL-10 production pathways (NES = 1.715, NOM *p* = 1.38 × 10⁻⁵, FDR q = 1.21 × 10⁻²) and IL-2/STAT5–associated signaling (NES = 1.699, NOM *p* = 2.96 × 10⁻⁵, FDR q = 2.11 × 10⁻³). Notably, the PD-1^high CD8⁺ T-cell exhaustion signature, strongly enriched in the FCGS disease state, was not enriched in the treated group. Importantly, although cytokine-regulated exhaustion pathways (e.g., IL-6/JAK/STAT3, IL-10 signaling) remain active after treatment, the loss of the PD-1^high exhaustion signature implies a meaningful reduction in terminal exhaustion pressure and may reflect improved T-cell functional potential relative to the pre-treatment state.

### Increased expression of CTLA4 + t cells in lymph nodes from FCGS affected cats

**Fig. 4:**
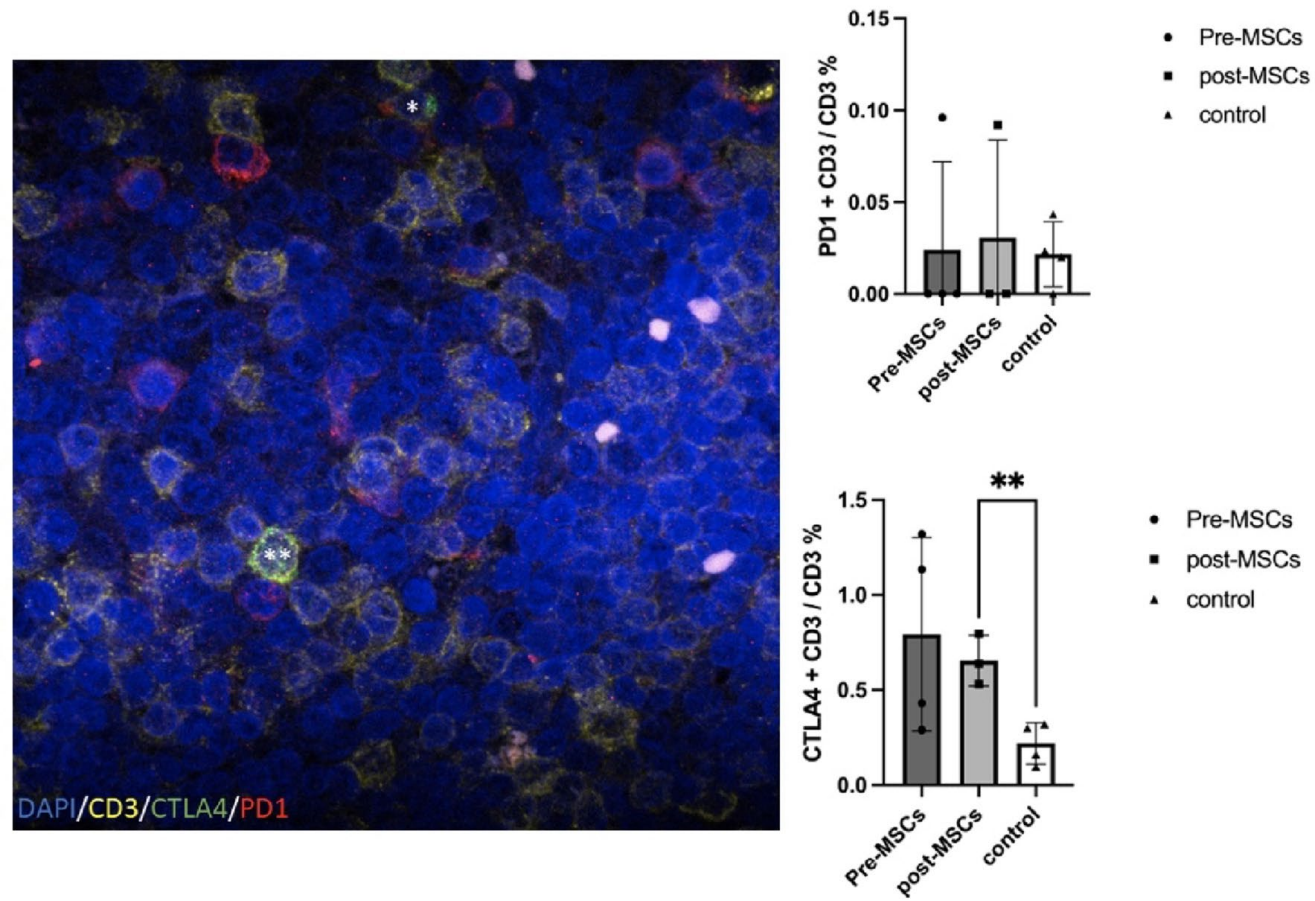
Immunofluorescence Analysis Identifies Persistent CTLA-4⁺ T-Cell Enrichment in FCGS Cats Pre- and Post-MSC Treatment. Immunofluorescence (IF) characterization of immune checkpoint expression in lymphoid tissues from FCGS cats before and after therapy. Representative IF image shows CD3⁺ T cells (green), CTLA-4 expression (yellow), PD-1 expression (red), and DAPI (blue). Quantification of checkpoint-positive T cells demonstrated similar proportions of PD-1⁺CD3⁺ cells across groups. In contrast, CTLA-4⁺CD3⁺ T cells were significantly elevated in FCGS cats pre- and post- therapy compared with healthy controls, suggesting sustained immunoregulatory signaling despite clinical improvement.

**Fig. 5:**
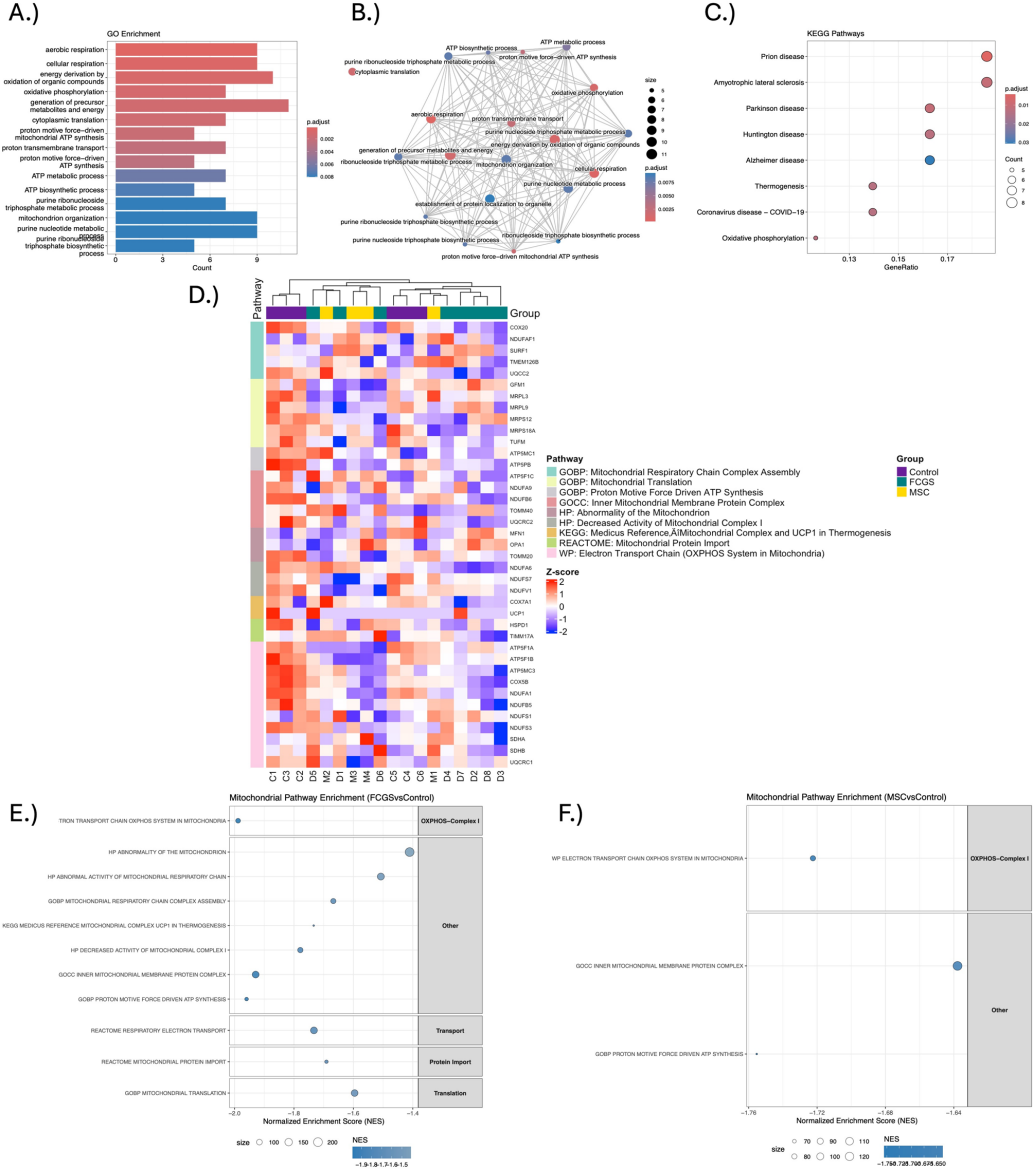
Mitochondrial transcriptional reprogramming in CD8⁺ T cells associated with FCGS. (**A**) GO enrichment highlights coordinated dysregulation of mitochondrial energy metabolism. (**B**) GO term network demonstrates strong functional clustering among mitochondrial and ATP-related processes. (**C**) KEGG pathway analysis shows enrichment of oxidative phosphorylation, thermogenesis, and neurodegeneration-associated pathways, reflecting shared signatures of mitochondrial stress and metabolic reprogramming. (**D**) Heatmap of curated mitochondrial gene sets shows broad downregulation across respiratory chain complexes, proton motive force–driven ATP synthesis, mitochondrial translation, and protein import machinery in FCGS cats (D and M). (**E**) GSEA for FCGS vs. control demonstrates strong negative enrichment of OXPHOS complexes, decreased mitochondrial activity, and impaired membrane and transport functions. (**F**) Treatment vs. control enrichment indicates fewer dysregulated pathways, with persistent but attenuated alterations in OXPHOS and mitochondrial protein import, consistent with partial metabolic reconditioning following therapy.

Given the enrichment of exhaustion-associated genes in cats with FGCS, we wanted to determine if canonical inhibitory receptors are upregulated in T cells within the oral lesions and the draining nodes (i.e. mandibular lymph nodes) of cats with FCGS. We stained formalin fixed and paraffin embedded (FFPE) lymph node and gingival tissues from cats with FGCS, prior and after treatment, as well as healthy controls with fluorescent antibodies against CD3, CTLA-4 and PD-1. While CTLA-4 + cells were commonly noted and were mostly CD3 + T cells, PD-1 expression was less common and most often was expressed on CD3- cells (Fig. 5). When comparing the frequency of CTLA-4 and PD-1 expressing CD3 + T cells between diseased and healthy control cats, we identified an increase in CTLA-4 + T cells in cats with FCGS pre-treatment and post-treatment cats (*P* = 0.0049). However, the difference between the pre-treatment FCGS cats and post- treatment cats was not statistically significant. The frequency of PD-1 expressing CD3 + T cells was not different in FCGS cats pre- and post-treatment, when compared to the control group (Fig. 4).

We observed T-cell infiltration in the superficial mucosa and submucosa of FCGS cats. The distribution of T cells in the mucosa tissue of FCGS cats was uneven. Some areas of affected tissue exhibit clusters of CD3 + cells, whereas other regions show a sparse, low-density distribution of these cells. There was no significant co-localization of CTLA-4, and PD-1 overlap with CD3 double-positive cells observed in the mucosal tissues (data not shown).

### Mitochondrial transcriptional dysregulation in FCGS and MSC-treated CD8⁺ T cells

To explore mitochondrial involvement in the immunopathogenesis of FCGS, we performed pathway-level analyses of CD8⁺ T cells from our three cohorts: healthy controls (C), cats with active disease (D), and treated cats (M) (Fig. 5). Gene ontology enrichment of differentially expressed genes revealed coordinated dysregulation of mitochondrial energy metabolism in FCGS CD8⁺ T cells. GO biological processes showed significant enrichment for pathways related to aerobic and cellular respiration, oxidative phosphorylation, proton motive force–driven ATP synthesis, mitochondrial protein import, and purine nucleotide metabolism, indicating broad impairment of mitochondrial bioenergetics (Fig. 5A). Network analysis of enriched GO terms demonstrated strong functional interconnectivity among processes governing ATP metabolic activity, mitochondrial organization, cytoplasmic translation, and energy derivation through oxidation of organic compounds, highlighting the central role of signatures associated with mitochondrial dysfunction in shaping the transcriptional landscape (Fig. 5B). KEGG pathway analysis similarly identified enrichment for oxidative phosphorylation, thermogenesis, and multiple neurodegenerative disease pathways (Parkinson’s, Alzheimer’s, Huntington’s, and prion disease), a pattern consistent with conserved signatures of mitochondrial stress, impaired electron transport chain function, and altered redox metabolism (Fig. 5C). Together, these enrichment profiles indicate that CD8⁺ T cells in FCGS exhibit a pronounced shift toward metabolic insufficiency and mitochondrial perturbation, reinforcing the link between chronic immune activation, transcriptional signatures associated with mitochondrial dysfunction, and a T-cell exhaustion-like state.

Curated mitochondrial pathway analysis revealed a broad suppression of mitochondrial gene expression in CD8⁺ T cells from FCGS cats, with genes spanning respiratory chain complexes, proton motive force–driven ATP synthesis, mitochondrial translation, and protein import machinery consistently downregulated relative to healthy controls (Fig. 5D). Pathway-level enrichment further confirmed this pattern: FCGS CD8⁺ T cells demonstrated strong negative enrichment across OXPHOS complexes (I–V), decreased respiratory chain activity, impaired mitochondrial membrane potential, and reduced protein import and translation (NES range approximately − 1.3 to − 1.6), indicating pervasive transcriptional signatures associated mitochondrial dysfunction (Fig. 5E). In contrast, treated cats exhibited a markedly attenuated signature, with only a limited set of pathways—primarily OXPHOS Complex I, inner mitochondrial membrane protein complexes, and proton motive force–driven ATP synthesis—remaining negatively enriched (Fig. 5F). The overall reduction in the number and magnitude of dysregulated pathways after therapy suggests partial metabolic recovery and improved mitochondrial function, consistent with the intermediate expression patterns observed in the heatmap.

## Discussion

In this study, we explored the immunopathogenesis of FCGS through transcriptomic profiling of CD8⁺ T cells. Most cats with FCGS harbored chronic viral infections implicating viral persistence as a potential trigger for sustained immune activation. RNA sequencing of CD8⁺ T cells revealed a shift from naïve toward effector and exhausted-like transcriptional programs, consistent with chronic antigenic stimulation and T cell dysfunction. Gene set enrichment analyses further confirmed enrichment of exhaustion- and terminal effector–associated pathways, alongside evidence of impaired mitochondrial function in untreated disease and partial restoration post-treatment. Together, these findings support a model of FCGS driven by chronic viral immune stimulation, T cell exhaustion-like, and mitochondrial dysregulation in the immune cell compartment.

Our bulk RNA-sequencing analysis of CD8 + T cells in FCGS cats revealed an exhaustion-like gene signature, akin to chronic viral stimulation seen in models like lymphocytic choriomeningitis virus (LCMV). The LCMV infection model is a classic and widely recognized model of chronic infection that provides a convincing reference for the gene expression map of CD8 + T cell exhaustion^4,41–43^. Notably, cats with FCGS showed upregulation of markers including EOMES, PRDM1 (Blimp-1), RGS16, CD244, CCRL2, CCR5, CCL5, TNFSF10 (TRAIL) and KLF2. Although effector genes such as GZMA, GZMB, and GZMK were upregulated in FCGS-affected cats, this likely represents a paradoxical state in which CD8⁺ T cells are transcriptionally primed for cytotoxicity but remain functionally impaired. While GZMF was not overexpressed in our dataset, prior research in tumor-infiltrating lymphocytes (TILs) identified GZMF expression specifically in PD-1⁺/TIM-3⁺ CD8⁺ T cells, a phenotype associated with terminal exhaustion. That study also proposed GZMF as a marker of terminally differentiated CD8⁺ T cells exhibiting heightened self-directed cytotoxicity^8^. Consequently, the co-expression of exhaustion-related and cytotoxic genes observed in our cohort could support a dysfunctional effector phenotype, in which immune cells are no longer capable of effective pathogen clearance despite retaining a cytotoxic gene signature or alternatively, GZM upregulation could represent a pathway for clearance of exhausted cells in FCGS patients.

Though our sample size was small, the effector vs. exhaustion comparison revealed a limited restoration of the effector gene expression in the post-treatment group. Expression levels failed to reach those observed for the healthy group. This might suggest that treatment primarily resolve the dysfunction of effector cells rather than revert them to a quiescent naïve state. At the same time, exhaustion is not exclusive to effector T cells; both effector and memory CD8⁺ T cells can become exhausted, although through different mechanisms and with varying degrees of functional impairment^44^. Our bulk RNAseq approach did not identify between these groups of cells hence the effects seen may be diluted. Additionally, our findings may influence by the fact that the vast majority (> 90%) of circulating CD8 + T cells in healthy cats are naïve cells, suggesting that exhausted CD8 T cells are very rare and that their molecular fingerprint would be diluted by naïve transcriptomic landscape^45^. Lastly, DGKA upregulation in healthy cats was unexpected but may reflect its physiological role in maintaining immune balance in cats^37,46^.

GSEA provided supporting evidence of transcriptional exhaustion in CD8⁺ T cells from cats with active FCGS. Compared to healthy controls, these T cells exhibited significant enrichment of gene sets associated with high PD-1 expression and terminal effector differentiation. Notably, though modest, the strongest enrichment was seen for the GSE26495_NAIVE_VS_PD1HIGH_CD8_TCELL_DN gene set, reinforcing the presence of PD-1–high, exhausted phenotypes. In contrast, cats post-treatment (M) showed a modest but strongest enrichment of gene sets associated with effector functions. The GOBP_INTERLEUKIN_10_PRODUCTION gene set reached statistical significance, suggesting some degree of functional recovery or reprogramming post-treatment. Together, these findings further support a model in which treatment may partially reverse CD8⁺ T cell exhaustion or restore effector function, but not fully restore these phenotypes to levels observed in healthy animals.

Furthermore, immunofluorescence staining for CTLA4 supported T-cell exhaustion-like phenotype in mandibular lymph nodes, although markers like PD1 and CTLA4 were not significantly elevated in the oral mucosa. These findings suggest that CTLA-4, rather than PD-1, may be the dominant inhibitory checkpoint pathway engaged within the lymphoid compartment of cats with FCGS. The persistent elevation of CTLA-4⁺ T cells before and after therapy indicates that immunoregulatory signaling remains active even after clinical improvement, potentially reflecting either sustained antigenic stimulation or a compensatory mechanism limiting tissue-damaging inflammation.

Although transcriptomic analysis demonstrated strong enrichment of a PD-1^high exhaustion signature in FCGS CD8⁺ T cells, PD-1 protein expression was not elevated in CD3⁺ T cells within lymph nodes or gingival tissues. This divergence likely reflects the multi-layered regulation of PD-1 expression, which is subject to post-transcriptional and post-translational control, as well as the heterogeneity of the CD3⁺ population analyzed by immunofluorescence, which encompasses non-CD8 T cells. Importantly, exhaustion is a heterogeneous state that can be driven by transcriptional and metabolic reprogramming independent of PD-1 surface expression. Indeed, key exhaustion-associated transcription factors and genes — including EOMES, PRDM1, RGS16, and CD244 — were markedly upregulated at the RNA level, and CTLA-4 was significantly elevated at the protein level in lymph node T cells, collectively supporting the presence of immunoregulatory and exhaustion-related signaling through alternative inhibitory pathways^12^. These findings highlight the complexity of exhaustion programs and emphasize the need to evaluate multiple regulatory axes beyond PD-1 when characterizing chronic T-cell activation in FCGS. We therefore use ‘exhaustion-like’ throughout to indicate that our findings are consistent with, but do not constitute definitive functional proof of, exhaustion — which would ultimately require ex vivo functional assays for confirmation.

The absence of CTLA-4 and PD-1 positive T cells (CD3+) in the oral mucosa of both the active disease groups could be indicative of a status of reduced immune tolerance in this tissue^47,48^. This potentially indicates the disruption of the balance of immune responses in FCGS prior to clinical intervention. The lack of detected PD-1 and CTLA-4 expression in mucosal T cells may also be due to the specific localization of the tissue, limitations in the detection methods, or localized sampling of mucosal CD8 + T cells.

The majority of cats with active FCGS in our cohort had evidence of chronic FCV infection (71%), which is consistent with prior epidemiological data implicating persistent calicivirus shedding in the pathogenesis of this disease^31^. Chronic viral antigenic stimulation is one of the most potent drivers of CD8⁺ T-cell exhaustion across species, acting through sustained TCR signaling and upregulation of inhibitory pathways including the EOMES/PRDM1 transcriptional axis documented here^34^. It is therefore plausible that the exhaustion-like transcriptional signature we observe in FCGS CD8⁺ T cells reflects, at least in part, a response to chronic viral antigen exposure rather than — or in addition to — a primary inflammatory disease process. This interpretation is further supported by the enrichment of IL-6/JAK/STAT3 and interferon-related gene sets, pathways known to be activated during chronic viral infection^23^. Importantly, the post-treatment group showed loss of the PD-1^high exhaustion signature, which may reflect both the therapeutic effects of treatment and the reduction in viral antigenic burden following FME. These two mechanisms cannot be distinguished in the current cross-sectional design, and future studies employing longitudinal sampling with concurrent viral load monitoring will be essential to delineate their relative contributions.

It is important to explicitly consider that full-mouth extraction alone, independent of any MSC-mediated effect, may account for a substantial portion of the transcriptional changes observed in the post-treatment group. By eliminating the subgingival and supragingival microbiome, FME removes the primary source of chronic antigenic drive, and this reduction in sustained TCR stimulation could be sufficient to permit partial reversal of the exhaustion-like transcriptional program, downregulation of inhibitory checkpoint pathways, and recovery of mitochondrial gene expression^31^. Accordingly, the transcriptional shifts described here should be interpreted as consequences of the combined FME + MSC intervention, with the relative contribution of each component remaining undetermined; the framing of these changes as “MSC-associated” throughout this manuscript is a convenience of nomenclature rather than a mechanistic attribution.

CD8⁺ T cells showed broader, pathway-level mitochondrial reprogramming in gene ontology and KEGG analyses. Downstream GSEA revealed significant negative enrichment of OXPHOS complexes, decreased mitochondrial activity, and impaired membrane and transport functions in cats with active FCGS analogous to what has been reported in tumor microenvironments^11^. Importantly, these mitochondrial pathways were restored in CD8⁺ T cells from treated cats, indicating partial metabolic recovery in responders. This distinction suggests that transcriptional signatures associated with mitochondrial dysfunction may impair modulation contributing to persistent activation and loss of effector control though functional studies would be necessary for confirmation.

It is important to note that mitochondrial dysfunction in this study is inferred from transcriptomic data showing downregulation of OXPHOS complex components, ATP synthesis machinery, and mitochondrial import pathways. These findings are consistent with impaired mitochondrial bioenergetics as described in murine exhaustion models and human chronic infection, but do not constitute functional proof of mitochondrial dysfunction. Functional validation through bioenergetic profiling (e.g., oxygen consumption rate measurements, mitochondrial membrane potential assays) will be an important objective of future studies.

This study should be considered an initial, exploratory investigation. The sample sizes, while sufficient for transcriptomic profiling and pathway-level inference, limit statistical power for subgroup analyses and preclude adjustment for covariates such as age, viral status, or time since diagnosis. Validation in an independent, larger cohort — ideally with paired pre- and post-treatment samples from the same animals — will be essential to confirm and extend these findings. Given that bulk sequencing, transcriptomic signatures of exhausted cells may have been diluted and nuances may have been lost that single-cell sequencing may better revealed. Additionally, the lack of longitudinal samples limits our ability to fully assess the effects of the treatment on the T cell transcriptome. An important limitation of this study is that the post-treatment group (M) received a combined intervention consisting of full-mouth extraction (FME) followed by MSC therapy, making it impossible to attribute the observed transcriptional changes to either intervention independently. FME is itself a significant therapeutic step that eliminates the primary source of chronic antigenic stimulation (i.e., sub- and supragingival microbiome) and may independently drive resolution of CD8⁺ T-cell exhaustion signatures and partial restoration of mitochondrial pathways. Future studies employing single-intervention control arms, ideally with FME-only and MSC-only groups, will be necessary to delineate the relative contributions of each component. Throughout this manuscript, references to ‘MSC-associated’ or ‘treatment-associated’ changes should be understood in this context. Another notable limitation of our transcriptomic analysis is the high number of significantly regulated genes lacking functional annotation, denoted by LOC identifiers. These uncharacterized genes represented a substantial proportion of the top differentially expressed transcripts. The limited annotation of the feline transcriptome hinders full biological interpretation of these findings and constrains pathway-level insights. This highlights the need for continued investment in feline genomic annotation and functional genomics, which would enhance the utility of domestic cats as translational models for human disease and improve resolution in studies of mucosal immunopathology and regenerative therapies.

In conclusion, our results reveal profound mitochondrial transcriptional suppression in CD8⁺ T cells during disease, including downregulation of Complex I activity, oxidative phosphorylation, and protein import pathways, features that closely mirror the metabolic and transcriptional hallmarks of T cell exhaustion described in human chronic viral infections and tumors. These cells also exhibited elevated expression of canonical exhaustion markers and transcriptional regulators, further supporting a dysfunctional effector phenotype. Therapy partially restored expression of both mitochondrial and immune effector pathways, suggesting that metabolic rescue may contribute to the reversal of CD8⁺ T cell exhaustion and underlie the observed clinical benefit. Taken together, our findings identify CD8⁺ T-cell exhaustion as a likely key immunological hallmark of FCGS and highlight mitochondrial reprogramming as a central mechanism underlying both disease pathogenesis and therapeutic response. These findings position FCGS as a naturally occurring, immunocompetent, and highly tractable model for studying the interplay between transcriptional signatures associated with mitochondrial dysfunction and CD8⁺ T cell exhaustion in chronic mucosal inflammation. Future studies employing single-cell sequencing of CD8⁺ T cells and longitudinal functional assays in healthy and FCGS-affected cats will be essential to build upon the targeted analyses presented here and to more fully delineate the cellular and molecular pathways driving this complex disease.

## Materials and methods

### Case selection

We collected blood and tissue samples, including oral mucosa and mandibular lymph nodes, from client-owned cats with a history of FCGS at the William R. Pritchard Veterinary Medical Teaching Hospital at the University of California in Davis, CA. Cat owners signed consent forms, and the study was approved under Institutional Animal Care and Use Committee (IACUC) protocols 22,738 and 21,766. Cats included in the study exhibited a clinical diagnosis of FCGS, characterized by bilateral inflammation affecting the caudal oral mucosa, specifically the palatoglossal folds^22^. Additional features included varying degrees of ulcerative, proliferative, or erythematous lesions extending to other oral sites such as the alveolar and buccal mucosa, as well as the gingiva with concurrent evidence of periodontal disease, tooth resorption or both. Affected cats demonstrated moderate to severe oral pain, as evidenced by clinical signs such as decreased appetite, dysphagia, drooling, pawing at the mouth, and reduced grooming or social behaviors. Diagnosis was histopathologically confirmed and characterized by a lymphoplasmacytic infiltrate and required persistence of inflammation despite routine periodontal treatments with or without extractions of severely affected teeth. Cats were otherwise healthy based on physical examination, complete blood count, biochemistry profiles, and urinalysis. Corticosteroids and antibiotics were discontinued at least 14 days before sampling, while pain management with opioids was continued as needed. Twelve cats met strict inclusion criteria and were divided into two groups: FCGS before treatment (D; *n* = 8) and FCGS after treatment (M; *n* = 4) which included full mouth extractions (FME) and MSC treatment. For D cats, blood was collected prior to surgery. M cats had blood collected at least six months after undergoing treatments. Lymph node and mucosal tissues were collected from affected cats, prior and after treatment. Blood, lymph nodes and mucosal tissues were also collected from 10 specific pathogen-free (C), non-FCGS cats which served as controls (Fig. 1; Table 1). Clinical evaluation, extraction treatment, and sample collection were performed by a board-certified veterinary dentist using standard probing and full-mouth intraoral radiography under general anesthesia. Anesthesia was supervised by a board-certified veterinary anesthesiologist. Anesthetic protocols were individualized to each patient but generally consisted of premedication with benzodiazepines and/or opioids, induction with alfaxalone or propofol, and maintenance with isoflurane. The pre-treatment (D) and post-treatment (M) groups are comprised of different individual cats and do not represent longitudinal paired samples from the same animals. Accordingly, comparisons between D and M groups reflect population-level transcriptional differences associated with disease state and treatment history, rather than within-individual changes over time.

**Table 1:** Demographics of patient population. The population consisted of three groups: patients with active FCGS (D), patients with FCGS at least six months after full mouth extraction and mesenchymal stem cell therapy (M), and SPF non-FCGS patients (C). DMH, domestic medium hair cat. DSH, domestic shorthair cat. F, female. M, male. MN, male neutered. FS, female spayed.

Case grouped no	Age (years)	Breed	Sex
PRE1	6	DMH	MN
PRE2	5	DSH	FS
PRE3	8	DMH	MN
PRE4	8	DSH	FS
PRE5	2	DSH	FS
PRE6	1.5	DSH	FS
PRE7	4	DSH	M
PRE8	5	DSH	FS
PRE9	2	DMH	MN
PRE10	2	DSH	F
PRE11	2	DSH	FS
PRE12	5	DSH	MN
PRE13	10	DSH	MN
PRE14	1	DSH	MN
POST1	15	DSH	MN
POST2	14	DSH	FS
POST3	14	DSH	FS
POST4	7	DSH	MN
POST5	7	DSH	MN
POST6	11	DSH	MN
POST7	3	DSH	MN
POST8	7	DSH	FS
POST9	9	DSH	MN
POST10	7	DSH	FS
POST11	11	DSH	MN
CON1	3	DSH	FS
CON2	2	DSH	FS
CON3	4	DSH	MN
CON4	8	DSH	FS
CON5	7	DSH	FS
CON6	4	DSH	FS
CON7	9	DSH	MN
CON8	5	DSH	MN
CON9	6	DSH	MN
CON10	1	DSH	F

### Viral testing

Infection with FCV was assessed by qRT-PCR on oral swab samples. Briefly, RNA was extracted from oral swabs using the QuantiTect Reverse Transcription Kit (Qiagen). cDNA synthesis was performed following the manufacturer’s protocol with modifications: gDNA was removed using gDNA WipeOut Buffer, and cDNA was synthesized using QuantiTect Reverse Transcriptase, RT Primer Mix, and Random Primers (Invitrogen). qPCR was performed using primers and probes specific for FCV (Supplemental material 1) and TaqMan Universal PCR Mastermix (Applied Biosystems). Reactions were run on a QuantStudio 7 Pro using standard cycling conditions. Cq values were determined using a threshold of 0.1 and baseline values of 3–15. he FCV test is 100% specific to known sequences in the NCBI database and can detect as few as 5–10 gene copies, demonstrating high sensitivity.

A commercially available enzyme-linked immunosorbent assay (ELISA) was used to screen for FeLV and FIV^49^. The FeLV test detects detecting the p27 core protein of the virus. The FIV test detects antibodies against FIV antigens. Twelve cats that were tested at their primary care vet typically underwent a point-of-care (POC) rapid antigen test for FIV and FeLV, facilitating immediate health management decisions.

### PBMC isolation and CD8 + T-cell enrichment

Whole blood samples were processed to isolate peripheral blood mononuclear cells (PBMCs) using a modified Hanks’ Balanced Salt Solution (HBSS) and Histopaque 1077 gradient exactly as previously described^50,51^. CD8 + T cells were enriched using a magnetic bead (Miltenyi Biotech) separation approach. We then labeled the isolated PBMCs with an anti-feline CD8 antibody (clone FE1.10E9) conjugated to a fluorescent dye and used a positive selection strategy, yielding 89.75 ± 4.65% CD8 + purity, confirmed by flow cytometry^52–54^.

### RNA extraction, library preparation, and sequencing

Enriched CD8 + T-cells were lysed in RLT buffer, and RNA was extracted using the RNeasy Mini Kit (Qiagen, Germantown, MD). RNA quantity and quality (ratios of 260/280 and 260/230) was measured with a NanoDrop spectrophotometer (Thermo Scientific). Samples with RIN scores > 7.4 were sequenced using the QuantSeq FWD kit (Lexogen). 3’-Tag sequencing was conducted on the Element Bio AVITI platform at UC Davis’s DNA Technology Core facility with single-end 100 bp reads. Reads were preprocessed using HTStream (https://s4hts.github.io/HTStream/), followed by alignment to the F. catus_Fca126_mat1.0 genome. Differential expression analysis between groups was conducted by the Bioinformatics Core at UC Davis using the limma-voom method from Bioconductor^55–57^.

### Transcriptomic analysis and differential expression

Reads were aligned to the Felis catus reference genome (F. catus_Fca126_mat1.0), and gene-level count matrices were generated from aligned reads. Downstream analyses were performed in R (v4.x). Raw count data were imported and processed using the edgeR and limma packages. Low-expression genes were retained for modeling, and normalization factors were calculated using the trimmed mean of M-values (TMM) method. Normalized counts were transformed using the voom function to model the mean–variance relationship prior to linear modeling. A design matrix was constructed based on experimental group (Control, FCGS, MSC), and differential expression was assessed using linear models implemented in limma, followed by empirical Bayes moderation of standard errors. Pairwise contrasts included FCGS vs. Control, MSC vs. Control, and MSC vs. FCGS. Differentially expressed genes (DEGs) were identified based on an adjusted P-value (false discovery rate, FDR) < 0.05. For visualization in volcano plots, an additional biological effect size threshold of |log₂ fold-change| > 0.25 was applied. DEG overlap between contrasts was assessed using gene identity-based Venn diagrams. Multidimensional scaling (MDS) was performed using the limma and vegan packages to visualize global transcriptional relationships between samples, with 95% confidence ellipses computed for each experimental group.

### Gene Set Enrichment Analysis (GSEA)

Gene set enrichment analysis was conducted using the fgsea package. Pre-ranked gene lists were generated based on signed log₂ fold-change values from the MSC vs. Control and FCGS vs. Control contrasts. Enrichment scores were calculated using permutation-based testing, and pathway significance was determined based on normalized enrichment scores (NES) and FDR-adjusted q-values. Gene sets related to mitochondrial function, immune activation, and CD8⁺ T cell phenotypes (naïve, effector, memory, and exhaustion states) were curated from publicly available databases and ortholog-mapped to Felis catus. Enrichment results were visualized using enrichplot and custom ggplot2-based workflows. Heatmaps of selected gene expression profiles were generated using the ComplexHeatmap package, with hierarchical clustering applied to both genes and samples based on scaled expression values. Color mapping was performed using circlize and RColorBrewer to enhance visualization of transcriptional patterns across CD8⁺ T cell subsets. All statistical analyses and visualizations were performed in R using tidyverse, ggplot2, and ggrepel. Venn diagrams were generated using the VennDiagram package. Figures illustrate transcriptional differences, DEG overlap, and pathway-level enrichment across control, FCGS, and MSC-treated groups.

### Immunofluorescence (IF) staining and imaging

Oral mucosa and lymph node tissue sections from FCGS and healthy cats were deparaffinized, rehydrated, and subjected to antigen retrieval^58^. Primary (CD3, CTLA-4 and PD-1) and secondary antibodies were applied, followed by nuclear counterstaining with DAPI (Supplemental material 2)^59^. Tissue sections were mounted and imaged using a Leica TCS SP8 STED 3X confocal microscope at 63x magnification. Images were analyzed to quantify PD1, CTLA4, and CD3 positive cells using ImageJ and QuPath. Variance in immunofluorescence (IF) staining between three groups was analyzed using One-Way ANOVA (or Kruskal-Wallis Test) with GraphPad Prism v10 and Excel.

## Data Availability

The raw RNA-seq data generated in this study has been deposited in the NCBI Gene Expression Omnibus (GEO; https://www.ncbi.nlm.nih.gov/geo/query/acc.cgi? acc=GSE328075) prior to publication and made publicly available. The GEO accession number is GSE328075. Additional data supporting the findings of this study are available from the corresponding author upon reasonable request.
